# Colombia a Source of Cacao Genetic Diversity As Revealed by the Population Structure Analysis of Germplasm Bank of *Theobroma cacao* L.

**DOI:** 10.3389/fpls.2017.01994

**Published:** 2017-11-21

**Authors:** Jaime A. Osorio-Guarín, Jhon Berdugo-Cely, Roberto Antonio Coronado, Yeny Patricia Zapata, Constanza Quintero, Gerardo Gallego-Sánchez, Roxana Yockteng

**Affiliations:** ^1^Centro de Investigación Tibaitatá, Corporación Colombiana de Investigación Agropecuaria – Corpoica, Cundinamarca, Colombia; ^2^International Center for Tropical Agriculture, Palmira, Colombia; ^3^Institut de Systématique, Evolution, Biodiversité-UMR-CNRS 7205, National Museum of Natural History, Paris, France

**Keywords:** SNP markers, integrated circuits of fluids, Fluidigm, molecular characterization, *Theobroma cacao*

## Abstract

Beans of the species *Theobroma cacao* L., also known as cacao, are the raw material to produce chocolate. Colombian cacao has been classified as a fine flavor cacao that represents the 5% of cacao world’s production. Colombian genetic resources from this species are conserved in *ex situ* and in-field germplasm banks, since *T. cacao* has recalcitrant seeds to desication and long-term storage. Currently, the collection of *T. cacao* of the Colombian Corporation of Agricultural Research (CORPOICA) has approximately 700 germplasm accessions. We conducted a molecular analysis of Corpoica’s cacao collection and a morphological characterization of some accessions with the goal to study its genetic diversity and population structure and, to select interesting accessions for the cacao’s breeding program. Phenotypic evaluation was performed based on 18 morphological traits and 4 biochemical traits. PCA analysis of morphological traits explained 60.6% of the total variation in seven components and 100% of the total variation of biochemical traits in four components, grouping the collection in 4 clusters for both variables. We explored 565 accessions from Corpoica’s germplasm and 252 accessions from reference populations using 96 single nucleotide polymorphism (SNP) molecular markers. Molecular patterns of cacao Corpoica’s collection were obtained amplifying specific alleles in a Fluidigm platform that used integrated circuits of fluids. Corpoica’s collection showed highest genetic diversity [Expected Heterozygosity (*H*_E_ = 0.314), Observed Heterozygosity (*H*_O_ = 0.353)] that is reduced when reference populations were included in the dataset (*H*_E_ = 0.294, *H*_O_ = 0.261). The collection was divided into four clusters based on population structure analysis. Cacao accessions from distinct groups showed some taxonomic concordance and reflected their geographic origins. For instance, accessions classified as Criollo were clearly differentiated in one group and we identified two new Colombian genetic groups. Using a number of allelic variations based on 87 SNP markers and 22 different morphological/biochemical traits, a core collection with a total of 232 accessions was selected as a primary genetic resource for cacao breeders.

## Introduction

*Theobroma cacao* L. also referred as cacao, is a native plant of tropical forests of South America (Motamayor et al., 2002) that belongs to the family Malvaceae. The Amazonian borders between Brazil, Peru, and the Southern Colombia encompass the highest genetic diversity of this tree species (Thomas et al., 2012). Today, cacao is grown with other fruit and commodity crops throughout the world in the humid tropics. Cocoa is the world’s third most important agricultural export commodity, after coffee and sugar. It provides economic benefits to some of the poorest areas of the world and it is the major foreign income for countries that dominate production such as Ivory Coast (Guiltinan et al., 2008). Besides the seeds producing the chocolate, cacao’s fruits are used to produce sweets, jellies, ice cream, liqueurs, cosmetic and medicinal products (Donald, 2004; Othman et al., 2007).

In Colombia, the cultivated area reported in 2014 was 160,276 hectares producing around 47,732 tons of cacao beans per year; placing it as the tenth producing country and area harvested worldwide; as well as the third in South America below Brazil and Ecuador. Since 1960, cacao’s Colombian production has not considerably improved, due in part to old cacao plantings, disease incidence and monoclonal planting (García-Cáceres et al., 2014). *Ex situ* germplasm banks have been created with the aim to support the survival of the species in its natural habitat and to conserve species diversity to be used in breeding programs (Guiltinan et al., 2008).

The bank of *ex situ* germplasm of the species *T. cacao*, guarded by the Colombian Corporation of Agricultural Research (CORPOICA), is located in two research centers (RC) placed in the Department of Santander (RC La Suiza) and Department of El Valle del Cauca (RC Palmira). The collection was initially created to find a solution to the problems of plant pathophysiology and production, exploring the Colombian diversity of cacao. Currently, the germplasm collection of Corpoica has been partially characterized morphologically and agronomically (Ballesteros et al., 2015). This characterization based mainly on phenotypic characters could be directly influenced by environmental factors, multigenic inheritance, quantitative inheritance and partial dominance of some characters. In order to assess cacao’s genetic diversity, it is necessary to characterize the collection using techniques that are not directly influenced by the environment. Different molecular markers have been developed and implemented to characterize cacao germplasm collections, such as microsatellites (SSR) by Lanaud et al. (1999), Motamayor et al. (2008), and Thomas et al. (2012). A first assessment of 100 accessions of Corpoica’s collection based on molecular markers (isozymes, restriction fragment length polymorphism (RFLP), random amplification of polymorphic DNA (RAPDs), and simple sequence repeats (SSR) showed an adequate genetic diversity (Sánchez et al., 2007).

A reduced and informative set of single nucleotide polymorphism (SNP) markers was selected as useful to analyze the genetic diversity and population structure of cacao based on expressed sequence tag (EST) data (Argout et al., 2008). A new technology called integrated circuits of fluids (IFC) using a platform based on a microwell plate-base system has been used to genotype cacao’s collection using the reduced set of SNP markers (Singh and Singh, 2015; Cosme et al., 2016; Motilal et al., 2016). The advantage of this technique is the lower running cost, the high throughput per run and a simplified setup of reactions (Xu, 2016). Diversity analyses using this technology indicated a high genetic diversity of the traditional varieties of cacao from Honduras, Nicaragua, and Puerto Rico, with an appealing potential for further studies on intrapopulation variation (Singh and Singh, 2015; Cosme et al., 2016; Motilal et al., 2016).

The aim of the present study was to determine the genetic diversity of 565 accessions of cacao from Corpoica’s collection based on SNP markers using Fluidigm. In order, to reach this purpose we will determine the genetic variability and population structure among accessions, and select accessions based on the diversity results to create a core collection that would constitute the primary resource for Colombian genetic breeding program.

## Materials and Methods

### Plant Materials

A total of 565 accessions of Corpoica’s Genebank (450 accessions) and a breeding collection (115 accessions) (Supplementary Table S1) were evaluated. Accessions are currently maintained *in vivo* at the research centers La Suiza (7°22′12″N 73°11′39″W) and Palmira (3°30′41″N 76°19′19″W) of the Colombian Agricultural Research Corporation (CORPOICA). Young or adult leaves of one individual were sampled by accession; and kept in hermetically sealed bags, containing about 100 g of silica gel. Finally, the plant material was transported to Molecular Genetics Laboratory at the research center Tibaitatá (4°41′45″N 74°12′12″W), where it was conserved at -80°C for further DNA extraction.

### DNA Extraction

Total DNA was isolated from young leaves using a modified CTAB extraction protocol for latex-containing plants (Michiels et al., 2003). The high levels of polysaccharides and polyphenolic compounds in cacao leaves can affect the DNA concentration, for that reason in some samples we used the DNeasy Plant Mini Kit (QIAGEN, Germany) according to manufacturer’s instructions. NanoDrop 1000 spectrophotometer (NanoDrop Technologies, Wilmington, DE, United States), was used to quantified total DNA. DNA samples showing absorbance ratios above 1.8 at 260/280 nanometre (nm) and above 1.2 at 260/230 nm were used for marker analysis. Additionally, the DNA quality was inspected using amplification with the following primers: (NS7) Forward 5′- GAGGCAATAACAGGTCTGTGATGC-3′ and (NS8) Reverse 5′-TCCGCAGGTTCACCTACG GA-3′ corresponding to 18S ribosomal RNA gene (White et al., 1990). The DNA was diluted to a working concentration of 20 ng/μL.

### SNP Genotyping and Data Processing

A set of 96 SNP markers, evenly distributed in the 10 cacao linkage groups, was used in this study. The linkage group and SNP position information is based according to the consensus map reported by (Allegre et al., 2012) (Supplementary Tables S2, S3). The selection of the SNP panel was based on the screening using Illumina’s GoldenGate Assay (Michel Boccara, unpublished data) and the reports in previous research on cacao (Ji et al., 2013; Fang et al., 2014; Lukman et al., 2014). Genotyping was performed using the Fluidigm 96.96 Dynamic Array IFCs (Fluidigm, San Francisco, CA, United States) according to the manufacturer’s protocol (Wang et al., 2009). Specific target amplification (STA) was performed prior to SNP genotyping analysis to allow the enrichment of template molecules for each individual integrated fluidic circuit (IFC) facilitating the multiplexing. PCR was performed in a 5-μL reaction containing at least 20 ng of the DNA sample according to the manufacturer’s protocol. Thermal cycling conditions were 15 min at 95°C, followed by 14 cycles of a two-step amplification profile of 15 s at 95°C and 4 min at 60°C. SNP type assays were performed using STA products diluted 1:20.

After a two-step incubation at 70°C for 30 min and 25°C for 10 min, a 5-min denaturation at 95°C was performed. Then, thermal cycling was carried out at 95°C for 15 s, 64°C for 45 s and 72°C for 15 s with a touchdown of -1°C per cycle from 64 to 60°C, followed by additional (24, 29, 34, or 39 cycles to allow the group conformation) of 95°C for 15 s, 60°C for 45 s and 72°C for 15 s. Endpoint fluorescent images of the 96.96 IFC were acquired at 28, 33, 38, and 43 cycles on an EP1^TM^ imager, and data was recorded with BioMark^TM^/EP1^TM^Data Collection Software (Fluidigm, San Francisco, CA, United States). Fluorescence plots obtained for each SNP were analyzed using the Fluidigm SNP genotyping analysis software. Genotype data was sorted according to the call rate percentage in order to establish a threshold for genotyping success and finally a matrix of genotypes vs. loci was created. A dataset of 252 samples provided by Dapeng Zhang (USDA) representing different cacao germplasm groups was included as reference populations, some of them have been analyzed and reported in previous studies (Ji et al., 2013; Cosme et al., 2016) (Supplementary Table S1). The dataset including only Corpoica’s accessions was named “Corpoica” and the dataset including Corpoica’s accessions and reference populations was named “Consense.”

### Population Structure and Cluster Analysis

The neutrality test of SNP markers was calculated using Tajima’s D (Tajima, 1989) by MEGA software v7.0.26 (Kumar et al., 2016). The estimation of the sub-populations number in both dataset, a Bayesian model of clustering analysis was carried using the software Structure v2.3.4 (Pritchard et al., 2000) with the following parameters: number of populations (*K*) set from 1 to 14, repeated 10 times, with a burn-in period of 200,000 iterations and 100,000 Markov Chain Monte Carlo (MCMC) repeats. The optimum *K* was selected by the method described by Evanno et al. (2005), using Structure Harvester (Earl and VonHoldt, 2011). The software Clumpp v1.1.1 (Jakobsson and Rosenberg, 2007) was used to line up the cluster labels (*K* selected) across runs and to estimate the degree of congruence between independent runs. Visualization of the results was done with Distruct v1.1 (Rosenberg, 2003). The assignation of each accession in a determinate cluster was established with a probability upper than 0.6; samples that presented the same probability for all clusters were not assigned in a specific cluster. Additionally, in order to recover the reference populations reported by Motamayor et al. (2008), the sample size of each reference population in the Consense dataset was simulated to 100 samples using the software Oncor (Kalinowski et al., 2007a). The analyses described above were also implemented in this simulated dataset.

The number of sub-populations of the most probable *K* for Corpoica and Consense datasets was confirmed with a principal coordinates analysis (PCA) using GenAlex 6.502 (Peakall and Smouse, 2012) and a cluster analysis using Neighbor Joining (NJ) method performed with Phylip 3.2 (Felsenstein, 1989) and viewed with FigTree software 1.4.2 (Rambaut, 2014). An analysis of molecular variance (AMOVA) and Wright’s *F* statistics parameters (*F*_IS_, *F*_IT_, and *F*_ST_) were conducted using the program Arlequin 3.5 (Excoffier and Lischer, 2010). The simulated dataset was not used for these analyses because the diversity statistics could be biased by the number of samples of the reference populations.

### Phylogenetic Analysis

Concatenated alignments using the sequence of the assay for each sample were created and used for the phylogenetic analyses for Consense dataset. Maximum likelihood (ML) bootstrap tree was constructed using PhyML 3.0 program (Guindon et al., 2010) implemented in the South of France bioinformatics platform^1^ for 1000 bootstrap replicates.

### Genetic Diversity

The genetic diversity results were carried out according to the population structure (most probable *K*). The allele frequencies, observed heterozygosity (*H*_O_), expected heterozygosity (*H*_E_), and polymorphism information content (PIC) were performed with GenAlex 6.502 (Peakall and Smouse, 2012) and Cervus 3.0.7 (Kalinowski et al., 2007b).

### Phenotypic Data

Phenotypic evaluation of the cacao collection was carried out at the Research Center La Suiza. One hundred and forty one accessions were evaluated for morphological characteristics, based on UPOV’s squash descriptor list (UPOV, 2011). Morphological descriptors were evaluated in six leaves, 5 flowering stems and 10 fruits per accession (**Table 1**). Additionally, biochemical traits (**Table 1**) were evaluated on 94 accessions at the chromatography – mass spectrometry laboratory from the Industrial University of Santander. Theobromine and caffeine contents were determined from an aqueous, degreased and filtered extract obtained from 80 to 100 g of moist cocoa beans using a high-performance liquid chromatography (HPLC). The Folin–Ciocalteu method (Kaur and Kapoor, 2002) was used to measure the total phenolic content using 100 g of moist cocoa grains. First, the extract was degreased with n-Hexane and polyphenols were extracted using a mixture of ethanol-acetone solvents and concentrated by rotoevaporation (Kaur and Kapoor, 2002). Mean values and standard deviation for quantitative data and mode values for qualitative data were calculated. In XLSTAT software version 2017 (Xlstat, 2017), the mean and mode values were used to conduct a principal component analysis (PCA) and clustering analysis (CA) using the Euclidean distance and clustering Ward method.

**Table 1:** Description of the qualitative and quantitative morphological variables recorded in 141 and 94 accessions of *Theobroma cacao*, respectively.

Trait		Coding	Characteristic	Code
Qualitative	Leaf	1–3	Blade size	LF-BS
		1–4	Blade shape of base	LF-BSB
		1–3	Blade shape of apex	LF-BSA
		1–6	Flush leaf color	LF-LC
	Fruit	1–5	Shape	FR-SH
		1,3,5,7	Basal constriction	FR-BC
		1–4	Shape of apex	FR-SA
		1,3,5	Surface	FR-S
		3,5,7	Exocarp thickness	FR-ET
		1–3	Seed shape in longitudinal section	FR-SLS
	Flower	1–3	Anthocyanin of pedicel	FL-AP
		3,5,7	Length of sepal	FL-LS
		3,5,7	Width of sepal	FL-WS
		1–4	Anthocyanin of sepal	FL-AS
		1-3	Color of lugula	FL-CL
		1–4	Anthocyanin of staminode	FL-AST
	Fruit	3,5,7	Seed length	FR-SL
		3,5,7	Seed width	FR-SW
Quantitative	Theobromine			TBR
	Caffeine			CA
	Polyphenols			PPS
	Ratio theobromine/caffeine			REL-TBR/CA

## Results

### SNP Genotyping

From the initial 96-SNP panel chosen to study genetic diversity, 87 SNP markers generated high call rates (>90%) across *T. cacao* samples from Corpoica (Supplementary Table S2). A total of 536 samples from the 565 evaluated accessions had a SNP call rate percentage higher than the threshold (>90%), when the filtrated set of 87-SNP were used. Sixteen outgroup individuals from the species *T. bicolor* and *T. grandiflorum* (Copoazu) (Supplementary Table S1) and 13 accessions from *T. cacao* were removed from the analyses because they had a low call rate (∼70%). Reference populations and Corpoica’s accessions shared a total of 78 SNP markers and were used for the analyses in the Consense dataset (Ji et al., 2013; Cosme et al., 2016).

### Population Structure and Cluster Analysis

**FIGURE 1:**
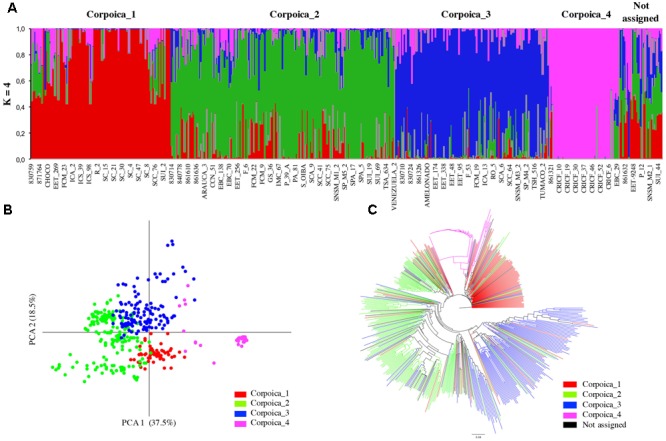
Inferred population structure of the *Theobroma cacao* from Corpoica collection. **(A)** STRUCTURE bar plot for *K* = 4, **(B)** principal coordinates analysis, **(C)** NJ-tree based on Nei’s genetic distances.

In this study, the value of Tajima’s *D* test was 2.47 indicating an excess of intermediate frequency alleles that can result from demographic processes such as population bottlenecks, population subdivision or migration (Maruyama and Fuerst, 1984). In order to corroborate population subdivision, genotypes of 536 samples were used to perform the population structure analysis. The mean posterior probability [lnP(D)] approach, used to find the number of sub-populations of cacao collections, fluctuated continuously and never reached a plateau (data not shown). In contrast, the Δ*K* analysis provided by the Evanno method (Evanno et al., 2005) suggested that the Corpoica dataset can be divided into four clusters (*K* = 4) (Supplementary Figure S1A). Most of the clusters obtained using Corpoica dataset consisted of a mix of accessions of different origin. The cluster named Corpoica_1 consisted mostly of accessions (90) from Colombia (SC, ICA, SCC, FCM, etc.), with 29 accessions from Trinidad, Ecuador, Mexico and Costa Rica. Corpoica_2 and Corpoica_3 clusters (134 and 75, respectively) consisted mostly of accessions from (Ecuador, Mexico, Peru, United States, Trinidad, Costa Rica, and Venezuela). In contrast, Corpoica_4 cluster consisted of Criollo accessions (CRICF and CR) all collected in Colombia (**Figure 1A**).

**FIGURE 2:**
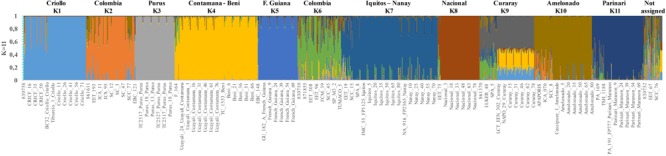
Inferred population structure of the *T. cacao* from Consense dataset with simulated reference populations. STRUCTURE bar plot for *K* = 11. The *K* = 2 and *K* = 6 are populations from Colombia.

The Δ*K* analysis obtained using the Consense dataset with the simulated data showed that the most probable population number is *K* = 4; however, peaks *K* = 7 and *K* = 11 were also identified as probable population numbers (Supplementary Figure S1B). For *K* = 4, the populations are mostly congruent with the groups obtained with the Corpoica dataset (Supplementary Figure S2A). Consense_1 cluster regrouped only accessions from Corpoica collection of different countries (Colombia, Peru, Mexico, Ecuador, and Costa Rica). Consense_2 cluster regrouped reference accessions from Iquitos, Nanay and Parinari with the accessions from Peru of the Corpoica germplasm bank. Samples from Amelonado and Guiana group were also included in the cluster Consense_2. Cluster Consense_3, consisted of reference accessions from Curaray, Nacional and EET from Ecuador, Beni from Bolivia, and Contamana from Peru and Purus from Brazil. In the cluster Consense_4, 100% of the CRICF accessions were grouped with the Criollo reference population confirming their classification as Criollo genotype. The value *K* = 11 was mostly congruent to the geographic distribution and the genetic backgrounds reported previously by (Motamayor et al., 2008) (**Figure 2**). The first cluster was exclusively composed by Criollo samples. The second cluster included most of the samples collected in Colombia. The third cluster was composed of individuals of Purus genetic background. The fourth cluster grouped samples from Contamana and Beni. The fifth cluster included samples from French Guiana. The sixth cluster contained samples with a high degree of admixture, mostly samples from Colombia and some accessions from Ecuador. The cluster 7 grouped accessions from Iquitos and Nanay Peruvian genetic backgrounds. The clusters 8 and 9 were composed of individuals from Nacional and Curaray Ecuadorian genetic backgrounds, respectively. Finally, clusters 10 and 11 grouped Amelonado and Parinari Brazilian genetic backgrounds.

The analysis of molecular variance (AMOVA) for the most probable sub-populations, indicated that the genetic variation for both datasets mainly occurred within individuals, accounting for 80% (Corpoica) and 52.75% (Consense), of the total variation, whereas the genetic variation among populations was 25.98 and 32.26%, respectively (**Table 2**).

**Table 2:** Summary of statistics of Analysis of Molecular Variance (AMOVA) for *Theobroma cacao* L. germplasm bank of Corpoica including reference genetic groups (Consense) and without them (Corpoica).

Analysis	Source of variation	Variance components	Percentage of variation (%)	*F*-statistics	*p*-value	*Nm*^a^
Corpoica	Among populations	5.21	25.98	*F*_ST_ = 0.259	0.00000	–
	Among individuals within populations	–1.21	–6.03	*F*_IS_ = -0.081	1.00000	–
	Within individuals	16.05	80.04	*F*_IT_ = 0.199	0.00000	–
	Total_Corpoica	20.06	100	–	–	0.46
Consense	Among populations	6.25	32.26	*F*_ST_ = 0.322	0.00000	–
	Among individuals within populations	2.90	14.97	*F*_IS_ = 0.221	0.00000	–
	Within individuals	10.23	52.75	*F*_IT_ = 0.472	0.00000	–
	Total_Consense	19.39	100	–	–	0.52

*^a^*Nm*: Gene flow or Number of migrants.*

Genetic differentiation (*F*_ST_) values of Corpoica collection varied from 0.143 (between population 2 and 3) to 0.520 (between population 2 and 4), indicating moderate differentiation among populations. Genetic differentiation (*F*_ST_) values for the Consense population are slightly higher than Corpoica collection ranging from 0.214 to 0.540; reference populations increased the differentiation between populations (**Table 3**).

**Table 3:** Pairwise genetic differentiation (*F*_ST_) values between subpopulations of *Theobroma cacao* L. germplasm bank of Corpoica including reference genetic groups (Consense) and without them (Corpoica).

Analysis	Subpopulation	Corpoica_1	Corpoica_2	Corpoica_3	Corpoica_4
**Corpoica**	Corpoica_1	0.000	–	–	–
	Corpoica_2	0.157^∗^	0.000	–	–
	Corpoica_3	0.190^∗^	0.143^∗^	0.000	–
	Corpoica_4	0.350^∗^	0.520^∗^	0.393^∗^	0.000
**Consense**	Consense_1	0.000	–	–	–
	Consense_2	0.236^∗^	0.000	–	–
	Consense_3	0.294^∗^	0.214^∗^	0.000	–
	Consense_4	0.460^∗^	0.540^∗^	0.430^∗^	0.000

*^∗^*p* < 0.00000.*

The inbreeding coefficient within populations (*F*_IS_) per locus was -0.081 for Corpoica and 0.221 for Consense. These results indicated an excess of heterozygotes in Corpoica’s collection, usually explained by the occurrence of outbreeding (random mating). However, the positive *F*_IS_ for the Consense population could indicate an increase in non-random mating added by the reference populations (**Table 2**). Furthermore, the inbreeding coefficient of an individual relative to the total population (*F*_IT_) was 0.199 for Corpoica dataset and 0.472 for Consense dataset (**Table 2**). The *F* statistics suggested the presence of different degrees of introgression among reference populations.

The NJ and PCA analysis were carried out for confirmation of sub-populations (**Figure 1B**). The PCA analysis showed that the first two coordinates explained 56% of the total variation within the Corpoica collection. No clear clustering was found compared to populations found in Structure, except for the consistent group formed by Criollo accessions. NJ analysis showed similar results of PCA analysis where the most differentiated group was comprised of Criollo accessions (**Figure 1C**). The Criollo accessions provide potential sources of differentiated genes for breeding programs. Similar results for PCA and NJ analysis were found for the Consense data set (Supplementary Figures S2B,C).

### Phylogenetic Analysis

**FIGURE 3:**
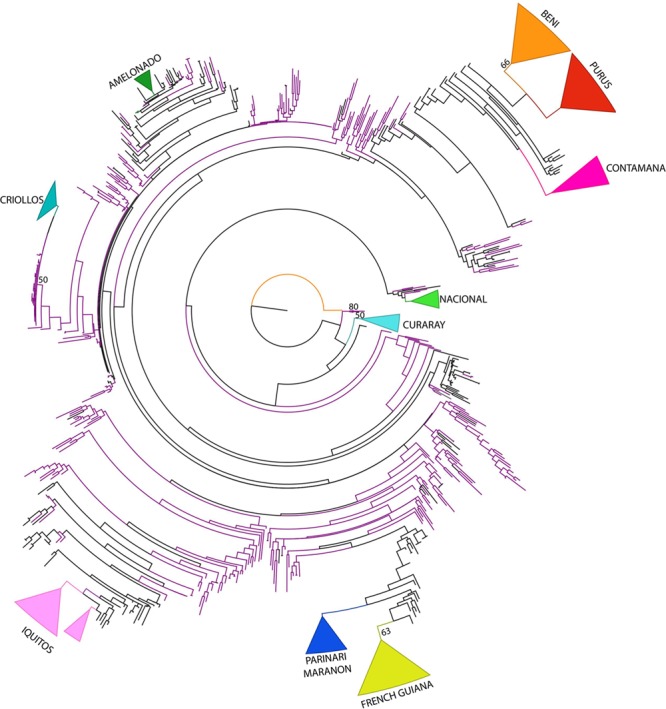
Maximum Likelihood Phylogenetic tree of *T. cacao* from Consense data. Bootstrap values higher than 50% are shown in nodes. Violet branches indicate accessions from the Colombian germplasm.

The ML phylogenetic analysis was conducted using Consense dataset. Bootstrap values are very low indicating low support for the nodes of the ML tree. However, the phylogenetic analysis could in general recover the cacao reference populations (**Figure 3**). In the ML tree, Colombian samples are distributed among branches of the tree indicating that Corpoica’s collection is diverse and has a good representation of different cacao genotypes. An individual from the species *T. grandiflorum* (Copoazu) was used as outgroup to root the tree.

The Curaray genetic group belonging to the Curaray Ecuadorian river had a basal position followed by the Nacional group also from Ecuador. A separate clade contained accessions from Ecuador (EET). A large clade containing the majority of accessions divided in two clades in which two Amazonian groups are distinguished. One regrouped Colombian accessions with the reference groups, Ucayali-Contamana, Purus from Peru and Beni from Bolivia, regions located at the South of Amazon Basin. In the second clade, four clades are distinguished one with the Upper Amazon reference groups (Parinari, Marañon, and Iquitos from Peru) regrouped with Colombian accessions, a second group with only Colombian accessions, and the last one that regrouped Criollos accessions and Amelonado accessions. As in previous analysis, Corpoica’s accessions classified as Criollo (CRICF and CR) were regrouped with the Criollo reference population supporting again their classification as Criollos. Colombian Criollos were collected in Cesar, North region of Colombia.

### Genetic Diversity

Summary statistics for the markers showed *H*_O_ values that ranged from 0.112 (for the TcSNP437 locus) to 0.550 (for TcSNP510 and TcSNP632 locus) and an overall *H*_O_ average of 0.371. Expected Heterozygosity (*H*_E_) ranged from 0.121 for the TcSNP1383 locus to 0.521 for the TcSNP915 locus and averaged 0.427. Polymorphic information content (PIC) ranged from 0.113 for TcSNP1383 to 0.460 for TcSNP709 (Supplementary Table S3).

The genetic analysis was carried out according with the population structure for each of the datasets. The diversity indices show that the *T. cacao* population from Corpoica used in the present study have a high level of genetic diversity with a mean value of *H*_E_ = 0.314 and *H*_O_ = 0.353, that are reduced when the reference populations are included (*H*_E_ = 0.294 and *H*_O_ = 0.261) (**Table 4**).

**Table 4:** Summary of genetic diversity of *Theobroma cacao’s* germplasm bank of Corpoica including reference genetic groups (Consense) and without them (Corpoica).

Analysis	Subpopulations	*N*^a^	*H*_O_^b^ (Mean ±*SD*)	*H*_E_^c^ (Mean ±*SD*)	*F*^d^ (Mean ±*SD*)
**Corpoica**	Corpoica_1	119	0.559 (0.042)	0.338 (0.024)	–0.477 (0.042)
	Corpoica_2	190	0.275 (0.014)	0.329 (0.017)	0.139 (0.018)
	Corpoica_3	131	0.494 (0.013)	0.427 (0.008)	–0.149 (0.017)
	Corpoica_4	59	0.040 (0.005)	0.077 (0.008)	0.348 (0.042)
	Not assigned	37	0.393 (0.018)	0.402 (0.014)	0.029 (0.024)
	Total_Corpoica	536	0.353 (0.013)	0.314 (0.009)	–0.030 (0.018)
**Consense**	Consense_1	134	0.447 (0.045)	0.336 (0.024)	–0.213 (0.065)
	Consense_2	307	0.226 (0.013)	0.314 (0.018)	0.255 (0.025)
	Consense_3	226	0.293 (0.014)	0.363 (0.014)	0.196 (0.028)
	Consense_4	77	0.048 (0.008)	0.055 (0.009)	0.075 (0.026)
	Not assigned	43	0.293 (0.017)	0.403 (0.013)	0.267 (0.036)
	Total_Consense	787	0.261 (0.012)	0.294 (0.010)	0.120 (0.020)

*^a^Number of samples, ^b^Observed Heterozygosity, ^c^Expected Heterozygosity, ^d^inbreeding coeficient; SD, standard deviation.*

At the subpopulation level, the highest genetic diversity was found in subpopulation Corpoica_1 (*H*_E_ = 0.559), whereas the lowest was detected for Corpoica_4 (*H*_E_ = 0.040). The highest genetic diversity for Consense subpopulations was found for Consense_3 (*H*_E_ = 0.363), whereas the lowest was detected for Consense_4 (*H*_E_ = 0.055) (**Table 4**).

### Phenotypic Data

Phenotypic evaluation was performed in 141 accessions for 18 qualitative traits related with morphological characteristics. The color of the leaf seems to be a distinguishing character because only 4.5% of accessions present shades of green. The predominant fruit shapes were the elliptical and oblong with 46 and 40%, respectively; only 14% corresponded to the obovate form. An important descriptor is the exocarp thickness that is associated with the size and number of seed that may contain the cacao fruit. It is expected that fruits having thin exocarp present big seminal cavity. From 141 accessions, 52% of the accessions presented a medium caliber of the exocarp and 18% of the genotypes presented a thin exocarp. The basal constriction is another important attribute to distinguish the cultivars, 15% does not present this characteristic. Slight constriction predominated in 47% of the evaluated genotypes, 34% of genotypes presented a moderate basal constriction and only 4% had a strong constriction. Seed length ranged from 1.85 to 2.86 cm and width from 0.75 to 1.56 cm (Supplementary Table S4).

**FIGURE 4:**
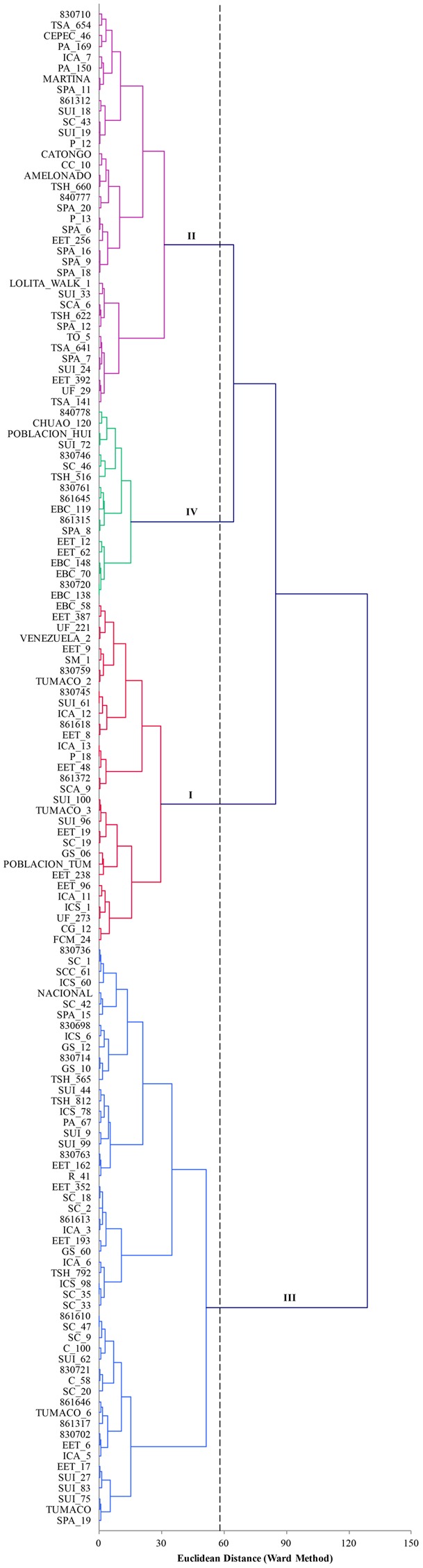
Plot of the first three components of PCA analysis of 18 morphological traits for 141 accessions of *T. cacao*. The hierarchical classification analysis grouped the accessions into four classes.

The first seven components of PCA analysis of morphological characterization explained 60.6% of the total variability. In total 18 components were needed to explained 100% of the variability. The first, second, and third components accounted for 15.1, 9.8, and 8.6%, respectively. The correlation of qualitative variables and its contributions were in order: anthocyanin of pedicel (0.760), seed length (0.691) and seed width (0.670). The cluster that grouped more accessions was cluster III (**Figure 4**), whereas cluster IV included only 18 accessions. Cluster I was characterized to present large and wide seeds with medium caliber of the exocarp. Accessions in cluster II were characterized by small and short seeds. In cluster II we found more accessions with thinner exocarp than that of those in cluster I. Accessions in cluster III were characterized to present medium longitude and width compared with the other groups. The species included in clusters IV, presented slightly wider exocarp and the seeds were almost as longs as the cluster I.

In terms of biochemical traits, 94 accessions for 4 quantitative traits were characterized. Theobromine ranged from 7.62 to 21.04 mg/g per dry sample. The caffeine content ranged from 0.67 to a maximum of 9.32 mg/g per dry sample. The ratio between theobromine and caffeine fluctuated from 1.69 to 25.42. Total polyphenols fluctuated between 8.66 and 46.13 mg gallic acid/g sample (Supplementary Table S5).

**FIGURE 5:**
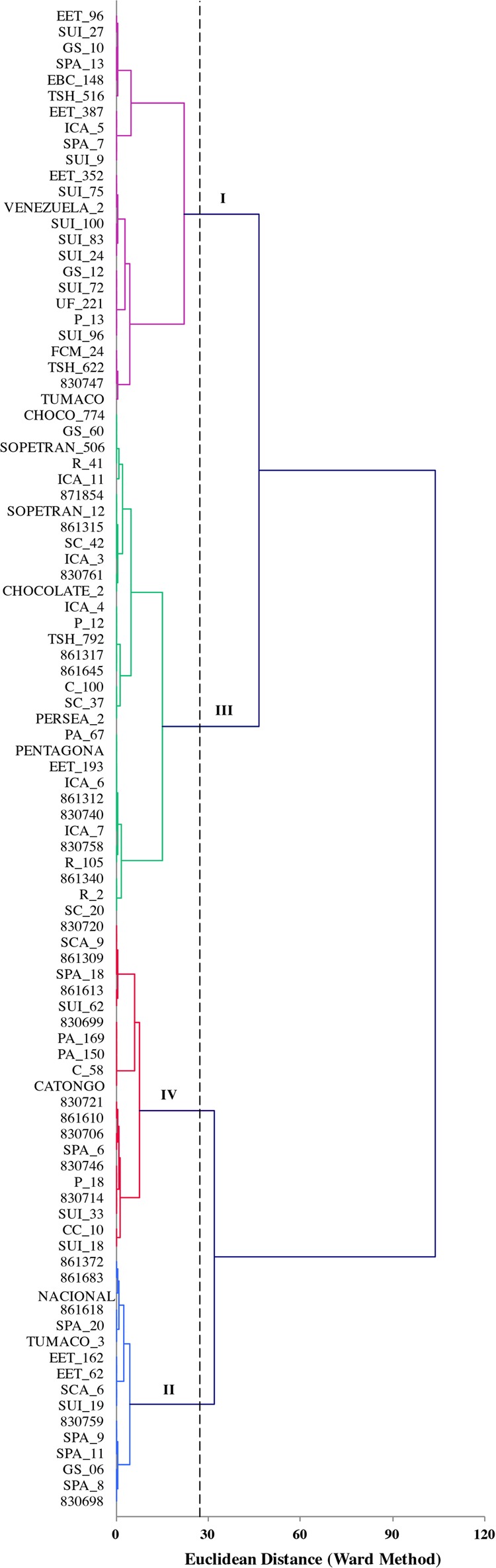
Plot of the first three principal components obtained from four quantitative variables measured in 94 accessions of *T. cacao*. The hierarchical classification analysis grouped the accessions into four classes.

The first four components of PCA analysis of biochemical characterization explained 100% of the total variability. The first, second, and third components accounted for 45.4, 33.5, and 15.5%, respectively. The correlation of quantitative variables was in order: caffeine (0.903), theobromine (0.774), and polyphenols (0.629). For the cluster analysis of the biochemical traits we used the first three components (**Figure 5**). Cluster I was characterized to present accessions with high levels of theobromine (>14 mg/g) and higher mean value of polyphenols (31.7 mg gallic acid/g). Accessions in cluster II were characterized by present lower levels of mean values of theobromine (10.9 mg/g), caffeine (2.4 mg/g) and polyphenols (22.4 mg gallic acid/g). Accessions in cluster III were characterized by high mean content of caffeine (5.12 mg/g), additionally similar results when we compared the mean value of theobromine (14.6 mg/g) and polyphenols (29.1 mg gallic acid/g) with clusters IV (theobromine 14.2 mg/g and polyphenols 29.8 mg gallic acid/g) were found. However, in cluster IV we found the best values concerned with relation between theobromine/caffeine.

The genetic groups identified using the molecular and phenotypic data were used to select the accessions that should conform the core collection. These accessions were distinct genetically from each other based on the results of the phylogenetic and structure analyses. Morphological and biochemical data was used to confirm the differentiation among individual of each genetic cluster. Additionally, the experience of the Corpoica’s cacao breeder and curator of the germplasm bank was also consulted to select materials for the conformation of this core collection.

## Discussion

Wide diversity of shapes and colors present in cacao plantations has been representing Colombian fine and aromatic cocoa since 1945. Targeted crosses between collected material from Amazonian and Trinitario materials were done to produce commercial hybrid materials that increase yield and resistance to the disease witches caused by *Moniliophthora perniciosa*. Since 2006, sampling effort done around the country have increased the germplasm conserved in genebanks and also permitted to define that cacao Criollo is clearly cultivated and native from the northern region of the country (Aranzazu et al., 2009).

Based on diversity studies and reconstruction of suitable habitats for the species, it is expected high cacao genetic diversity in Colombia (Motamayor et al., 2008; Thomas et al., 2012). The Amazonian regions of Peru, Colombia, and Ecuador have been considered the geographical origin of the species, because there are the regions with the highest genetic diversity of cacao (Thomas et al., 2012). So far, few studies using part of the germplasm available have explored Colombian cacao diversity (Sánchez et al., 2007; Ballesteros et al., 2015). The present study showed that 450 cacao accessions conserved in the National Germplasm Bank and 115 accessions from Corpoica’s breeding collection conformed a rich and diverse collection supported by molecular and phenotypic data.

Although studies based on SSR markers reported other cacao collections more diverse than Corpoica’s (Boza et al., 2013; Pokou et al., 2014; Bidot Martínez et al., 2015), it appears, more diverse when compared with studies based on SNP markers. According to Botstein et al. (1980), PIC can be used to evaluate the level of gene variation, the value is equal or greater than 0.5 which suggested high informative with a SSR marker loci. The PIC values for bi-allelic SNP markers range from 0 to 0.5, whereas for multi-allelic SSR markers, the PIC value can be as high as 0.5–1.0. However, for this reason, it is not possible to compare the results between SSR and SNP markers. For instance, level of gene variation of Colombian collection evaluated by PIC values (PIC > 0.4) is higher than values reported for Puerto Rico’s naturalized populations (PIC < 0.375) (Cosme et al. (2016). It also appears to be highly polymorphic when compared with other studies (Ji et al., 2013; Takrama et al., 2014; Cosme et al., 2016). Colombian accessions appeared to be highly diverse; Corpoica_1 cluster containing mostly accessions from Colombia presented the highest *H*_O_ = 0.559. An excess of heterozygotes was found in Corpoica’s collection, probably due to hybrid and foreign cacao germplasm (25% of foreign accessions). In contrast, a deficit of heterozygotes (*H*_O_ < *H*_E_) was found when reference populations were included probably because these populations are mostly homogenous, particularly Criollo and Amelonado well known by their highly homozygous genomes and their ability to self-fertilize (Argout et al., 2011; Motamayor et al., 2013). As confirmation of this hypothesis the population Consense_2 (Amelonado majority) and the population Consense_4 (Criollo majority) also presented deficit of heterozygotes (**Table 4**).

A molecular study based on SSR markers showed that cacao diversity is classified in 10 different genetic groups from the Upper Amazon, Lower Amazon, Orinoco and Guyana (Motamayor et al., 2008). Criollo genotype appeared as a separated group being the most genetically differentiated group. However, the present study could not recover the 10 genetic groups even when reference populations were included. The collection was divided only in four clusters with significant genetic variance among and within the populations revealed by AMOVA. It is probably necessary to include more markers in order to recover the reference clusters. However, Ji et al. (2013) reported that a reduced set of SNP markers (∼26 SNP) could provide 99.99% confidence to identify an individual cacao tree. In the present case, 87 SNP markers were used giving high confidence to the results. Another explanation could be that SNP markers are less powerful compared to SSR markers in terms of relative kinship estimation and population structure analysis (Van Inghelandt et al., 2010; Yang et al., 2011). In fact, SSR markers have higher allelic diversity than SNP markers (Filippi et al., 2015). A recent study using a similar set of SNP markers reported that Puerto Rican cacao fit into four (Criollo, Trinitario, Amelonado, and UAF) genetic backgrounds (Cosme et al., 2016). In contrast, this study identified two populations composed mostly by Colombian samples (**Figure 2**). This result would support the idea that Colombia has unique genetic backgrounds and is one of the diversity centers of cacao.

The phylogenetic tree recovered the recognized genetic groups and, Colombian accessions were distributed in clades with different reference groups, showing they were representative of cacao diversity. Different analyses indicated Criollo group as the most genetically differentiated group with the highest *F*_ST_ values (**Table 3**) as reported earlier by Motamayor et al. (2008). This result would support the subdivision of *T. cacao* in two morpho-geographic subspecies, ssp. *cacao* and ssp. *sphaerocarpum* (Cuatrecasas, 1964; de la Cruz et al., 1995), which correspond, respectively, to the two main genetic groups “Criollo” and “Forastero” (de la Cruz et al., 1995). Criollo group probably evolved in sympatry with Forastero populations but with reduced genetic flow and was introduced later by the man to Central America (Motamayor et al., 2013). However, further work to confirm the origin of Criollo genotype is necessary.

Corpoica accessions regrouped with Criollo reference population were mostly collected in the North of the country, in areas of influence of the Serrania del Perijá (Swisscontact, 2014) and Sierra Nevada de Santa Marta corresponding to geographical distribution of Criollos (Aranzazu et al., 2009). These cacao materials were collected from a region located in average conditions to 832 masl, 24.8°C of temperature and 73.4% humidity. Their adaptation to the conservation conditions from the research center La Suiza located at 530 masl, with even higher temperature and relative humidity, has hampered fruit and flower production. Thus, phenotypic characterization was difficult in those genotypes; only vegetative characteristics were recorded for few accessions. Those accessions present green tones of the leaves distinctive to the Criollo genotype (UPOV, 2011).

To establish core collections to be used in breeding programs, it is necessary to fully characterize the collection genetically and phenotypically. The SNP markers (87) used in combination with 22 phenotypic data were effective to construct a core collection with the aim to conserve phenotypic and genetic variability (Supplementary Table S1).

One important characteristic to select genotypes is the bean or seed size because it determines the lipid content, an important quality index for cocoa producers. In general, bean mean size of Corpoica’s collection measured in length (2.33 cm) and width (1.22 cm); low values compared to other studies. Santos et al. (2012) reported an average value for seed length of 2.72 cm and seed width of 1.40 cm for the Brazilian germplasm collection of the Centro de Pesquisas do Cacau (CEPEC). While Vásquez-Ovando et al. (2015) reported averages of 2.60 and 1.82 cm for seed length and width, respectively, for material collected in cacao farms in Mexico. Differences are probably due to the size of the sample collection; they used 15 and 45 accessions of *T. cacao*, respectively, compared to 141 accessions used in the present study. Variation in bean attributes (shape, length, width, thickness, and weight) in *T. cacao*, has been related to the genetic origin (Clement et al., 2003). For instance, Ballesteros et al. (2015) analyzing Tumaco native materials (South West region of Colombia) found in general small bean sizes; 1.78 cm and 1.16 cm for seed length and width, respectively. The variation in fruit characteristics could probably be a consequence of genetic differentiation or to the domestication process (Motamayor et al., 2002; Clement et al., 2010).

Chemical composition is also an important attribute to select genotypes because it determines cacao flavor (Kongor et al., 2016). Cacao beans are rich in polyphenols (about 15% of dry bean weight) (Krähmer et al., 2015) which confer astringent and bitter sensations and contribute significantly to the green and fruity flavors of cocoa liquors (Noor-Soffalina et al., 2009). One study from Venezuela using fermented cocoa from Mérida and Ghana from two localities found total polyphenol contents ranging from 24.09 to 53.23 mg gallic acid/g. In this study, we found polyphenol contents ranging from 8.7 to 46.1 mg gallic acid/g.

The ratio between theobromine and caffeine (T/C) is a measure associated to the quality of cacao and to the genotype. T/C values less than or equal to two correspond to Criollo type, values between two and six correspond to the Trinitario type, six to eight to miscellaneous and from eight onward to Forastero type (Davrieux et al., 2003; Zambrano et al., 2010; Aprotosoaie et al., 2016). Based on this ratio, Corpoica’s collection would mostly consist of Trinitario (69%) and miscellaneous cacaos (17%) with few accessions of Forastero type (9%) and Criollo type (3%). Highest ratios in Colombian accessions were from genotypes (830714, 830721) from South of Colombia (Nariño). Colombian accessions 830758, Chocolate_2 and Choco_774 from the Choco region (North Western Colombia), presented the lowest ratios and are closed related to the Criollo clade (**Figure 2**). This trend would support the denomination that 80% of Colombia’s cocoa is fine with aroma and flavor. Additionally, as noted earlier, most of Colombian accessions forming the Criollo group do not have chemical data because they did not produce fruits to make the analyses. Additionally, this result could indicate that exists an association between genotype and T/C ratio. Results from Trognitz et al. (2013) showed a relationship between T/C ratio and genotype in Nicaraguan cacao. Nevertheless, Trognitz et al. (2013) also inferred that T/C ratio and polyphenol content is influenced by post-harvest procedures.

## Conclusion

Establishing the genetic diversity of Colombian cacao collection will enhance the proper utilization of genetic resources. In the present study, based on population structure and morphological characterization, a core collection of cacao was constructed using 87 SNP markers and 22 different traits. This core collection will serve as a primary source for further genetic association and functional analyses for novel genes as well as for developing cacao’s breeding program. The results found in the present study suggest that, despite containing commercial materials, Corpoica’s collection has a significant level of genetic diversity. Based on the results of the present study, Colombia would have unique genetic populations and would be a center of cacao diversity.

## Author Contributions

JO-G and RY conceptualized and conceived the project and its components. JO-G, JB-C, YZ, and CQ carried out the IFC genotyping. JO-G, JB-C, and RY analyzed the data, wrote the manuscript and RY corrected and edited it. RC conducted the phenotypic evaluation. GG-S provided the genotyping infrastructure. All authors reviewed and contributed to draft the manuscript as well as read and approved the final manuscript.

## Conflict of Interest Statement

The authors declare that the research was conducted in the absence of any commercial or financial relationships that could be construed as a potential conflict of interest.

## Footnotes

1 http://www.atgc-montpellier.fr/phyml/
