# Supplementary material for: Colombia a Source of Cacao Genetic Diversity As Revealed by the Population Structure Analysis of Germplasm Bank of Theobroma cacao L
Source: Front Plant Sci. 2017 Nov 21;8:1994. doi: 10.3389/fpls.2017.01994 (PMC5702303; doi:10.3389/fpls.2017.01994)
Supplement: Supplementary file 6 [file Image_1.pdf]

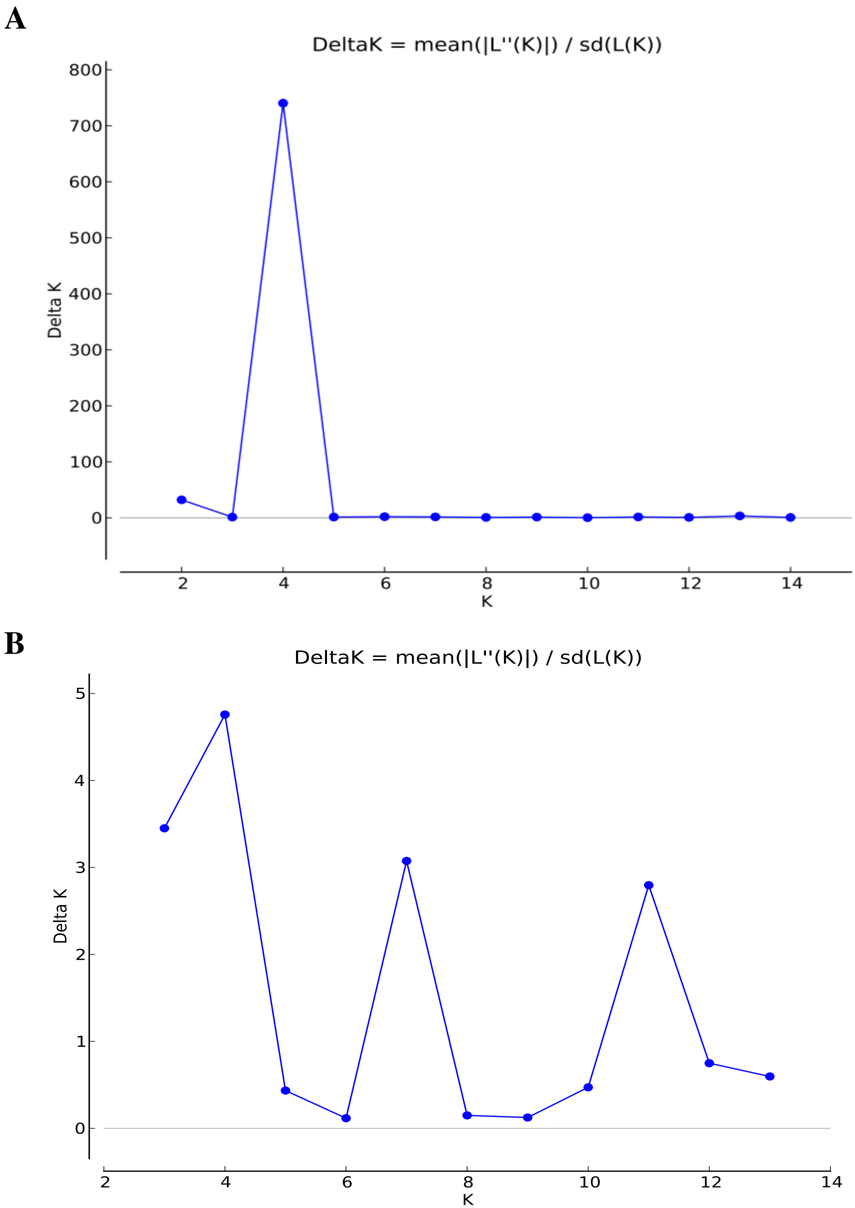

Figure S1: ΔK plots obtained from Evanno method **(A)** Corpoica's collection, **(B)** Consense population with simulated data for reference population. Graph of delta K values (y-axe) against assumed sub-populations (x-axe) showing the ideal number of groups present in the *Theobroma cacao* population using of 87 and 78 polymorphic SNPs, respectively. The highest peak is K=4 for both data sets.
