# Supplementary material for: Colombia a Source of Cacao Genetic Diversity As Revealed by the Population Structure Analysis of Germplasm Bank of Theobroma cacao L
Source: Front Plant Sci. 2017 Nov 21;8:1994. doi: 10.3389/fpls.2017.01994 (PMC5702303; doi:10.3389/fpls.2017.01994)
Supplement: Supplementary file 7 [file Image_2.PDF]

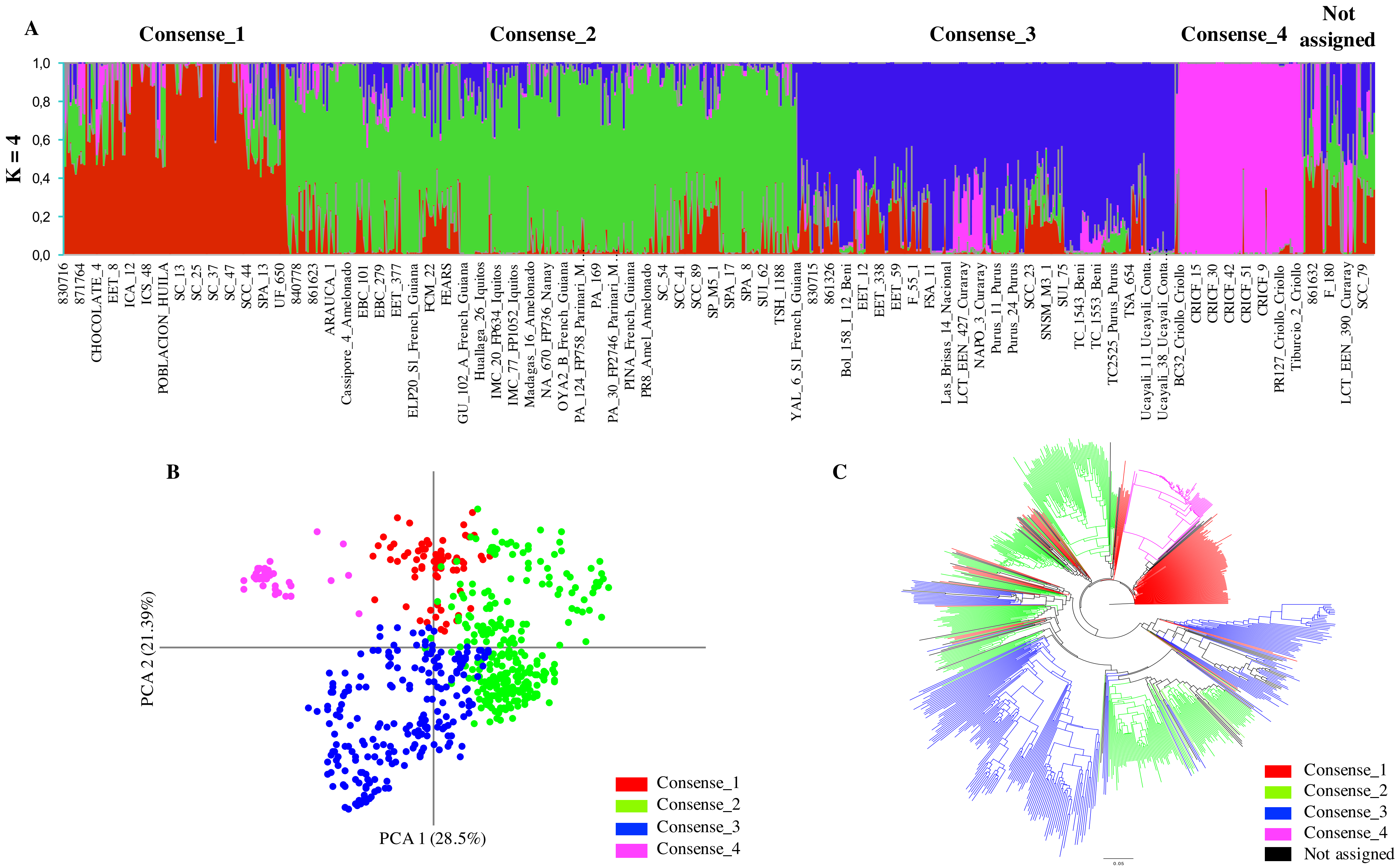

Figure S2: Inferred population structure of the *Theobroma cacao* collection using the Consense dataset. **(A)** STRUCTURE bar plot for K=4, (**B**) Principal Coordinates Analysis, **(C)** NJ-tree based on Nei´s genetic distances.
